# Long-Term Thermal Stabilization of Poly(Lactic Acid)

**DOI:** 10.3390/ma17112761

**Published:** 2024-06-05

**Authors:** Jannik Hallstein, Elke Metzsch-Zilligen, Rudolf Pfaendner

**Affiliations:** Division Plastics, Fraunhofer Institute for Structural Durability and System Reliability LBF, 64289 Darmstadt, Germany; jannik.hallstein@lbf.fraunhofer.de (J.H.); elke.metzsch-zilligen@lbf.fraunhofer.de (E.M.-Z.)

**Keywords:** poly(lactic acid), thermal aging, degradation mechanisms, hydrolysis stabilizer, antioxidants

## Abstract

To use polylactic acid in demanding technical applications, sufficient long-term thermal stability is required. In this work, the thermal aging of polylactic acid (PLA) in the solid phase at 100 °C and 150 °C is investigated. PLA has only limited aging stability without the addition of stabilizers. Therefore, the degradation mechanism in thermal aging was subsequently investigated in more detail to identify a suitable stabilization strategy. Investigations using nuclear magnetic resonance spectroscopy showed that, contrary to expectations, even under thermal aging conditions, hydrolytic degradation rather than oxidative degradation is the primary degradation mechanism. This was further confirmed by the investigation of suitable stabilizers. While the addition of phenols, phosphites and thioethers as antioxidants leads only to a limited improvement in aging stability, the addition of an additive composition to provide hydrolytic stabilization results in extended durability. Efficient compositions consist of an aziridine-based hydrolysis inhibitor and a hydrotalcite co-stabilizer. At an aging temperature of 100 °C, the time until significant polymer chain degradation occurs is extended from approx. 500 h for unstabilized polylactic acid to over 2000 h for stabilized polylactic acid.

## 1. Introduction

In order to meet the demands for increased sustainability, one scenario targets the replacement of conventional petro-based plastics by bio-based polymers. The most promising bioplastic today is polylactic acid (PLA). The starting monomers can be produced via fermentation from sugar cane, sugar beet or corn starch [1]. Due to its high rigidity and strength, it has the potential to act as a substitute for engineering plastics in durable applications [2,3]. In order to realize its potential, however, some hurdles regarding long-term stability must be overcome. Due to the chemical structure of polylactic acid, the polymer chains are susceptible to chain scission and have only limited long-term stability. They are, in particular, sensitive to hydrolytic degradation, but also to thermo-oxidative degradation. Hydrolytic degradation of polylactic acid has already been extensively investigated [4,5,6,7], and there are also studies on the natural weathering behavior [8,9]. Studies on thermo-oxidative degradation, however, have mostly been carried out above the melting temperature [10,11,12]. Little is known about its thermo-oxidative degradation and the associated degrading mechanisms in the solid phase, knowledge which is essential for the material’s application.

The most detailed investigations, particularly regarding the degradation mechanisms, were carried out by Rasselet et al. [13]. PLA films were examined in an oven aging process at temperatures between 70 °C and 150 °C and characterized with regard to changes in mechanical properties and molecular weight. An attempt was also made to clarify the exact degradation mechanism using Fourier-transform infrared spectroscopy. It was found that at temperatures above 100 °C, there is a significant reduction in molecular weight after just a few hundred hours. The mechanical properties also decrease accordingly. The study concludes that during aging in the solid phase at elevated temperatures, oxidative degradation primarily takes place, while hydrolytic degradation processes are negligible, although it was not possible to identify specific degradation products. Further studies on the thermal aging behavior of PLA-based compounds were carried out by Anakabe et al. [14]. Here too, the mechanical properties were investigated during oven aging at temperatures of up to 110 °C. They also found a drastic loss of properties after a few hundred hours at temperatures above 90 °C. Even at lower temperatures of 50 °C and 70 °C, the thermal stability is inadequate and after a few thousand hours, there is a significant reduction in the mechanical properties. Therefore, it can be concluded that pure PLA does not have sufficient thermal stability for long-term applications at elevated temperatures.

To be used in such applications, the polylactic acid must be stabilized. One strategy for this is the use of additives to improve the durability of the polymer. Based on previous findings on degradation during thermal aging, the addition of antioxidants could provide a remedy. These are, for example, sterically hindered phenols (primary antioxidants) or phosphites and thio-based compounds (secondary antioxidants) [15,16]. Primary antioxidants stabilize by donating a proton and deactivating radicals formed by oxidative attack on the polymer chain [17]. Secondary antioxidants act synergistically, converting the resulting hydroperoxide compounds into non-reactive products [18,19,20]. While phosphites are primarily effective at processing temperatures, the thio-based compounds are effective at lower temperatures (<150 °C), such as during thermal aging. The use of antioxidants as stabilizers in polylactic acid has, so far, mostly been investigated in studies on processing stability [12,21,22,23,24,25]. Amorin et al. [12] investigated primary and secondary antioxidants (phosphites) in multiple extrusion steps and examined the resulting molecular weight change over five extrusion steps. It was found that a 0.2% combination of a sterically hindered phenol and a phosphite was suitable for stabilizing PLA. However, it was also pointed out that further non-radical degradation reactions occur during processing, which are not stabilized by the addition of antioxidants. Oliveira et al. [21] also investigated a combination of primary and secondary antioxidants as stabilizers during long-term extrusion. They found that a good stabilizing effect was achieved during a 25 min extrusion by adding 0.2% of Irganox B900 (consisting of four parts phosphite and one part sterically hindered phenol). Cicero et al. [22] investigated tris (nonylphenyl)phosphite (TNPP) as a stabilizer during processing. The addition of 0.11 % and 0.8% TNPP without a primary antioxidant also ensured good stabilization. Furthermore, Sirishina et al. [23] and Polidar et al. [26] have shown that phosphites have a stabilizing effect during processing. However, both studies also observed that the addition of phosphites also catalyzes the hydrolytic chain scission of PLA, which would be disadvantageous for long-term applications.

The present work provides a new insight in the possible degradation mechanisms of PLA during thermal aging in the solid phase. Based on past studies, it can be assumed that thermo-oxidative chain cleavage is dominant, but clear evidence through the identification of potential degradation products could not be provided. Polymer chain scission due to hydrolysis cannot be ruled out either. For this purpose, unstabilized PLA was subjected to oven aging and the polymer chains were characterized using nuclear magnetic resonance spectroscopy. Based on the findings regarding the degradation mechanism, various stabilizers were compounded into PLA in a second step. The additivated PLA was again subjected to oven aging and changes in melt viscosity were examined over time to determine the efficiency of the applied additives.

## 2. Materials and Methods

### 2.1. Materials

**Figure 1:**
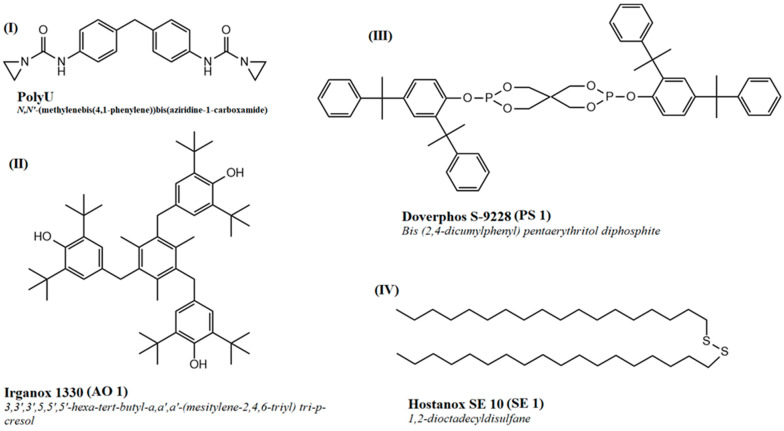
Chemical structures of used additives.

A commercially available PLA grade (Luminy L130, TotalEnergies Corbion, Gorinchem, The Netherlands) was used. This grade is a fully biobased, semicrystalline PLA grade with an l-isomer content of at least 99%. The high isomeric purity enables a good crystallization behavior and use in high-heat applications. As stabilizers, different commercially available additives were used. A bifunctional aziridine derivate (PolyU, Menadiona, Barcelona, Spain), an uncoated calcium hydrotalcite (Actilox CAH EXP 0213 (HTC 1), Nabaltec, Schwandorf, Germany), a primary phenolic antioxidant (Irganox 1330 (AO 1), BASF, Ludwigshafen, Germany), a secondary phosphite antioxidant (Doverphos S-9228 (PS 1), Dover Chemical, Dover, OH, USA) and a secondary antioxidant based on a thioether (Hostanox SE10 (SE 1), Clariant, Muttenz, Switzerland) were used as supplied by the manufacturers. The chemical structures of the used additives are shown in Figure 1.

AO 1 (Irganox 1330) is a phenolic antioxidant that is particularly suitable for linear polyesters. PS 1 (Doverphos S-9228) is a phosphite (secondary antioxidant) with high hydrolytic stability, as it is known that phosphitic structures can accelerate the degradation of PLA under certain environmental conditions [23,26]. SE 1 (Hostanox SE 10) is also a secondary antioxidant based on a thioether. The combination of primary and secondary antioxidants achieves particularly good stabilization against oxidative degradation, which is why the additives are not only tested alone but also in combination [19]. The amounts of additives added were at the usual commercial concentrations for antioxidants as well as hydrolysis stabilizers.

### 2.2. Compounding

Compounding was performed on a co-rotating parallel twin-screw extruder Process 11 (Thermo Fisher Scientific, Karlsruhe, Germany) with a screw diameter of 11 mm and an L/D ratio of 40. Prior to the compounding, PLA granules were cooled in liquid nitrogen and milled on a cutting mill (Rapid Granulier Systeme GmbH & Co. KG, Kleinostheim, Germany). Before the extrusion process, milled PLA was vacuum dried for 16 h at 80 °C to a moisture content of below 250 ppm. The calcium hydrotalcite was vacuum dried for 16 h at 150 °C. Other additives were used as supplied. Milled polymer and additives were premixed in a bag and added to the volumetric dosage unit, which was set to a mass throughput of 800 g per hour. The screw speed of the extruder was set to 200 rpm, and the temperature profile was set to increase from 170 °C in the feeding zone to 200 °C in the mixing zones and the die. Vacuum degassing, a water bath and a pelletizer were used.

### 2.3. Aging Tests

For thermal aging tests, around 120 g of compounded PLA granules were weighed in aluminium trays. The trays were punctured with small holes to ensure air circulation. The trays were placed in a heating chamber with forced convection (Binder M115, Binder GmbH, Tuttlingen, Germany), which was set to the aging temperatures of 100 °C and 150 °C, respectively. The lower temperature was selected with regard to the continuous operating temperature of semicrystalline PLA (~100 °C) to replicate the highest possible operating temperature, while the higher temperature, which was a few degrees below the melting point of PLA (~170 °C), was selected to achieve the highest rate of aging feasible. To ensure homogeneous aging, the trays were removed daily, and the granules were mixed thoroughly. In addition, the position of the trays in the oven was varied daily. Samples of 10 g of the granules were removed at different time intervals and sealed in vacuum bags until further testing.

### 2.4. Characterization

The melt volume rate (MVR) was measured on a mi2 melt flow indexer (Göttfert Werkstoff-Prüfmaschinen GmbH, Buchen, Germany) according to DIN EN ISO 1133-2 [27]. The temperature was set to 190 °C, and the stamp was loaded with a weight of 2.16 kg. The heating time was set to 4 min and about 8 g of granules were used to perform the tests. Prior to the MVR measurements, the pellets were dried in a vacuum oven at 80 °C for 16 h and sealed in vacuum bags until shortly before the start of the measurement to ensure no further polymer degradation during the heating time. For each removal time, two measurements per compound were performed, and the average values are reported.

Size exclusion chromatography (SEC) measurements were performed using SEC 1260 system by Agilant Technologies (Waldbronn, Germany), consisting of a degasser (G1322A), isocratic pump (G1310B), autosampler (G1329B), thermostat (G1316A), variable wavelength detector (G1314F), refractive index detector (G7800A), two Agilent-PLgel-MIXED-C columns and a PLgel guard column. Chloroform was used as the eluent (c = 2 g L^−1^) at 35 °C with a flow rate of 1 mL min^−1^. Calibration was performed using polystyrene standard (PSS Polymer Standards Service GmbH, Mainz, Germany) over a molecular weight distribution of 417–2,520,000 g mol^−1^. The elution curves were evaluated in a molecular weight range from 1500 g mol^−1^ to 300,000 g mol^−1^.

^1^H-NMR spectra were recorded on a Bruker NanoBay 300 spectrometer (7.05 T, Bruker, Ettlingen, Germany). About 15 mg of the polymer was dissolved in 0.7 mL CDCl_3_.

## 3. Results

### 3.1. Thermal Aging of PLA and Investigation of the Degradation Mechnism

In order to select the most suitable stabilizers for material development, it is necessary to understand how the polymer chains degrade. In a first step, the unadditivated PLA was subjected to thermal aging at 150 °C, and the chain degradation was observed at various intervals via the melt volume rate (MVR). The MVR is an indicator of the melt viscosity, which in turn correlates with the polymer chain length. Increasing MVR values mean a decrease in viscosity and, therefore, shorter polymer chains. The samples were subsequently analysed via nuclear magnetic resonance spectroscopy and size exclusion chromatography to draw conclusions about the degradation mechanism.

**Figure 2:**
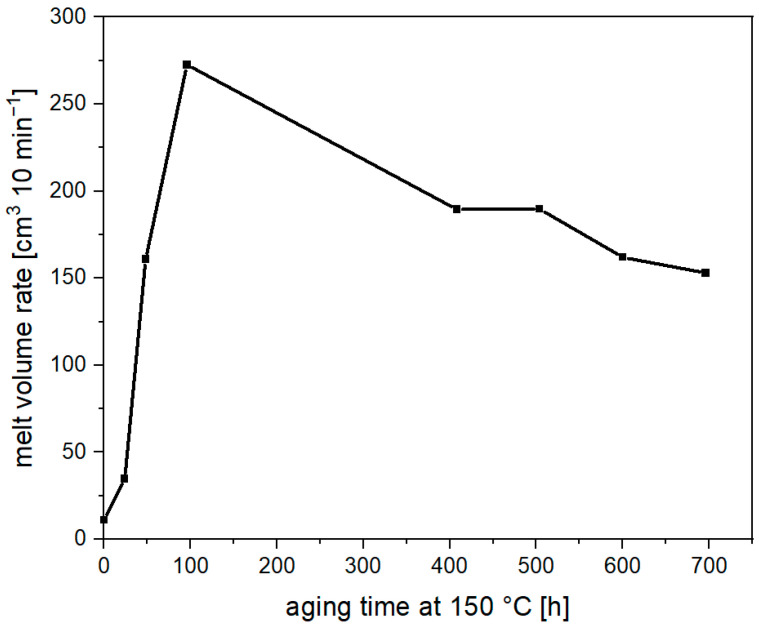
Melt volume rate of PLA (black line) during thermal aging at 150 °C.

Figure 2 shows the melt volume rate over the aging period at 150 °C. A significant increase in the MVR value can be observed after a very short time. After just 24 h, it has tripled (11 cm^3^ 10 min^−1^ → 35 cm^3^ 10 min^−1^). After 48 h, the value is already over 160 cm^3^ 10 min^−1^, and after just under one hundred hours, it is at around 275 cm^3^ 10 min^−1^. This shows that a rapid degradation of the material occurs at 150 °C and is in line with previous studies that have found a drastic drop in molecular weight after a relatively short aging period (<200 h) [13]. As the aging process progresses, however, a surprising behavior emerges. Contrary to expectations, there is no further increase in the MVR, which would have been expected as chain degradation normally proceeds. However, the MVR decreases during further aging, and after just under 700 h, it is at 150 cm^3^ 10 min^−1^, almost 50% below the maximum value. This indicates an increasing viscosity of the melt, which can be caused by two phenomena. The first is a molecular weight increase, either by solid phase condensation or by branching reactions, and the second is migration of oligomeric substances that previously acted as plasticizers during the MVR measurement and lead to an excessive decrease in viscosity. In order to verify these hypotheses, SEC measurements were carried out.

**Figure 3:**
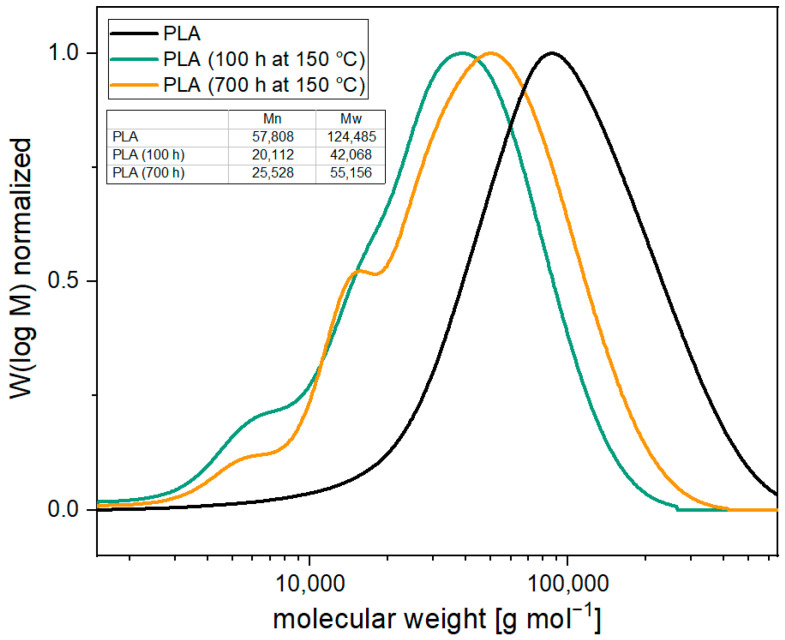
Molecular weight distribution of PLA over the course of the thermal aging at 150 °C.

**Figure 4:**
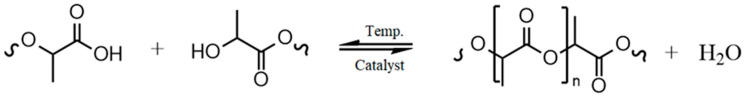
Polymerization reaction of PLA [29].

Figure 3 illustrates the molecular weight distributions as well as the number and weight average molecular weights at different aging intervals. The molecular weight is reduced by approx. 65% within 100 h. Observation of the molecular weight distribution shows that there is primarily a shift to lower molecular weights. This suggests that degradation occurs randomly along the polymer chain. The polydispersity index (PI = Mw/Mn) remains almost unchanged at 2.1. Nevertheless, a small shoulder can be detected at low molecular weights of less than 10,000 g mol^−1^. After 700 h of aging, the molecular weight increases again, as assumed by the MVR. The number- and weight-average molecular weights increase by 27.5–30%. A parallel shift towards higher molecular weights can be observed. The polydispersity index remains almost unchanged at 2.2. The molecular weight distribution shows that the shoulder decreases below 10,000 g mol^−1^, while a new shoulder appears at molecular weights of 10,000–20,000 g mol^−1^. This behavior indicates that the increase in molecular weight is caused by solid phase condensation, since primarily shorter chains recombine with each other (see Figure 4) [28].

In a branching reaction, a shoulder in the range of high molecular weights would have been expected, resulting in a significant increase in the polydispersity index. Although this behavior has not yet been observed in the thermal aging of PLA, it is known that PLA can polymerize in the solid phase under certain conditions [28,30,31]. Solid-phase polymerization is usually carried out at temperatures between 100 °C and 160 °C. In addition, a vacuum is often applied, and the samples are polymerized under nitrogen. This helps to remove moisture from the system and shift the reaction equilibrium, allowing molecular weights of well over 100,000 g mol^−1^ to be achieved in some cases [28]. Solid-phase polymerization is usually carried out for between 5 and 50 h, with the addition of a catalyst. As the granules are aged at 150 °C, the temperature is sufficiently high. It can also be assumed that residual catalyst (or its reaction products) from the original polymer synthesis is still present in the material. There is no vacuum or nitrogen flow in the oven, which is also reflected in the significantly lower changes in molecular weight compared to the literature [28,30,31]. The time periods during oven ageing, on the other hand, are significantly prolonged, with the drop in MVR occurring over a period of several hundred hours. For the condensation reaction to take place, a significant number of hydroxyl and carboxyl end groups are required to push the reaction equilibrium in the direction of esterification, and a catalyst and high temperatures are also necessary. The hydroxyl and carboxyl end groups can only be formed during the aging process, as it is known from the literature that the PLA grade used in this study has a very small number of carboxyl end groups [32]. To verify the formation of the required end groups, a ^1^H-NMR spectrum of the samples at different aging states was recorded and is shown below.

**Figure 5:**
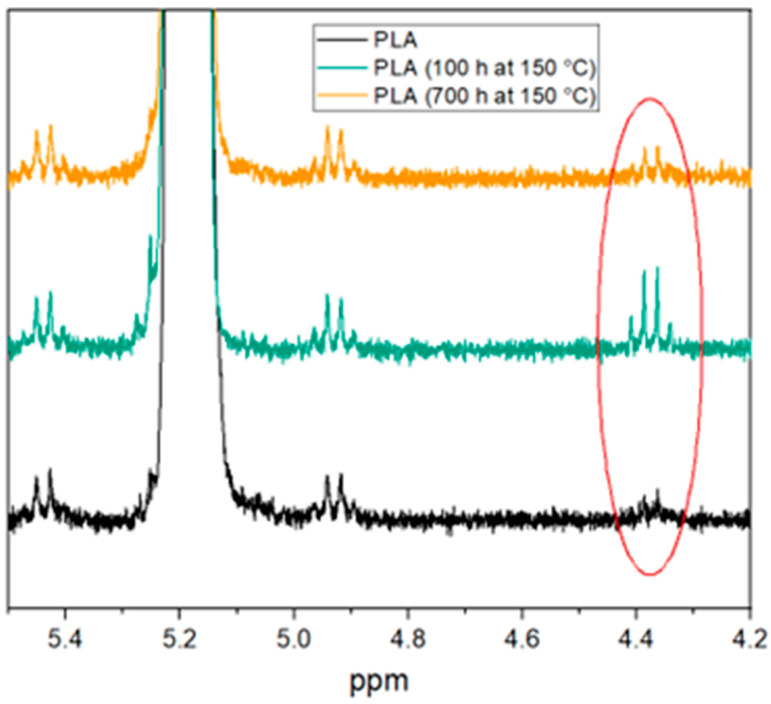
^1^H-NMR of the differently aged samples in the range from 4.2 to 5.5 ppm. Changes in the spectrum occur at a chemical shift of 4.4 ppm (red circle).

Figure 5 shows the ^1^H-NMR spectra of the samples after different aging times. Since a direct identification of the end groups was not possible, the proton of the CH unit in the main chain was selected instead, which is at approximately 5.2 ppm, as the presence of a hydroxyl or carboxyl end group shifts this signal [33]. Observation of the aging states shows that after 100 h of thermal aging, a pronounced signal appears at 4.4 ppm, which was not present for the unaged PLA. This suggests that degradation reactions occur during aging, during which, hydroxyl and carboxyl end groups are formed. After 700 h of thermal aging, the signal is significantly less pronounced, which is synonymous with the disappearance of these groups. This clearly indicates solid phase condensation reactions, which were already suggested by the results from the MVR and SEC measurements.

**Figure 6:**
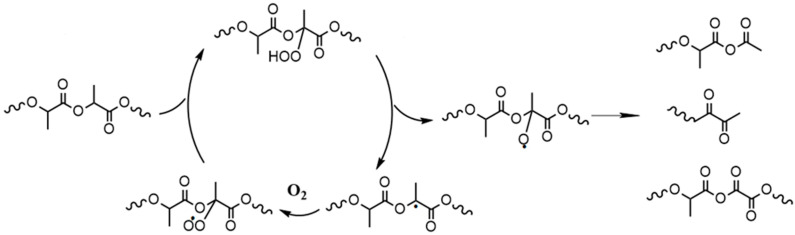
Oxidative degradation of PLA (according to [13,39]).

**Figure 7:**
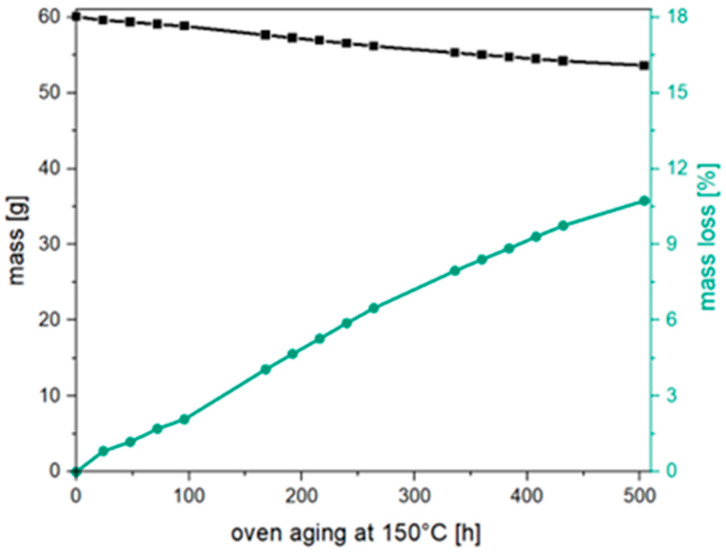
Mass (black line/squares) and percentage mass loss (green line/dots) of 60 g PLA granules during 150 °C aging.

However, based on the oxidative degradation mechanisms previously described in the literature during oven aging, no formation of hydroxyl or carboxyl end groups would be expected (see Figure 6). Therefore, it has to be assumed that other degradation mechanisms, in addition to oxidative degradation, take place. Hydrolytic degradation due to residual moisture in the material is also conceivable. Due to the high temperatures in particular, even small amounts of moisture could be sufficient to cause hydrolytic chain scission. However, during hydrolytic chain scission, in addition to the random chain breaks in the main chain, there is also frequent scission at the terminal ester bonds, which should also result in a significant number of low-molecular chains [34,35,36]. However, these could only be determined to a limited extent in the GPC and should have led to a change in the polydispersity index, which is not seen. One reason for this finding could be the migration of these oligomeric components out of the material. As the aging temperature of 150 °C is significantly higher than the glass transition temperature of approx. 60 °C, there is a high degree of chain mobility, which facilitates migration [37,38]. To investigate this, the weight loss of PLA granules was determined during oven aging. Therefore, 60 g of PLA granules were stored at 150 °C, and the weight of the granules was monitored over time. Figure 7 shows the weight and the relative weight loss over 500 h at 150 °C. A continuous loss of mass over the entire observation period is observed. The relative weight loss is already at over 1.5% after 100 h and at 11.5% after 500 h. This is a clear indication that there are not only random chain scissions but also that a significant number of degradation reactions at the end-units of the polymer chain occur, which leads to the formation of highly mobile lactic acid oligomers.

In conclusion, it can be stated that the thermal degradation of PLA in the solid phase does not primarily involve oxidative degradation mechanisms as assumed based on previous publications [13]. Furthermore, a significant number of chain scissions occur, leading to the formation of carboxyl and hydroxyl end groups, presumably triggered by hydrolytic chain scission.

### 3.2. Thermal Aging of PLA and Investigation of Possible Stabilizers

After the possible degradation mechanisms were investigated and identified, the PLA was compounded with additives and subjected to thermal aging. As the pure PLA was already severely degraded after just a few days at an aging temperature of 150 °C, the aging temperature was reduced to 100 °C. This corresponds to the continuous use temperature of semi-crystalline PLA and thus represents a possible application temperature. The additives used were a synergistic hydrolysis stabilizer combination consisting of an aziridine-based inhibitor (PolyU) and an acid scavenger (calcium hydrotalcite). The high stabilization efficiency of this combination against hydrolysis was demonstrated in a previous study [40]. On the other hand, commercially available antioxidants were also added to stabilize against possible oxidative degradation.

**Figure 8:**
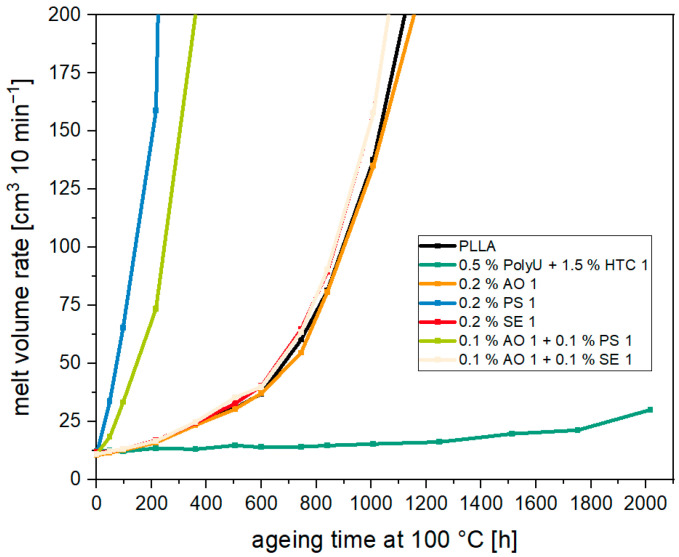
Melt volume rate over aging time at 100 °C for PLA with different stabilizers.

Figure 8 shows the MVR for various stabilized PLA compounds over 2000 h of oven aging at 100 °C. For the unstabilized PLA, it can be seen that there is a slow continuous increase in MVR at the beginning. The MVR doubled after approx. 360 h (11 cm^3^ 10 min^−1^ → 24 cm^3^ 10 min^−1^). This cumulative increase continues until 600 h (37 cm^3^ 10 min^−1^) before a sharp increase in the MVR occurs. The addition of phenolic and thio-based antioxidants alone and in combination does not result in a stabilizing effect. The MVR is equivalent to the unstabilized material, and after more than 600 h, there is a drastic increase in the melt volume flow rate. This is in line with literature findings which observed only a slight change in aging stability at 100 °C with the addition of 0.5% Irganox 1010 (phenolic antioxidant) [41].

When the phosphite stabilizer is added, however, the MVR increases drastically after even shorter aging times. For the sample with only phosphite, the MVR is already over 50 cm^3^ 10 min^−1^ after less than 100 h and over 150 cm^3^ 10 min^−1^ after 200 h. In combination with the phosphite stabilizer, the drastic increase in the MVR is only marginally delayed since the amount of phosphite is halved. The lack of a stabilization effect or the accelerated degradation by the antioxidants again suggests that chain degradation during oven aging is not primarily caused by oxidative degradation. In particular, the behavior of the samples stabilized with phosphite suggests that acid-catalyzed hydrolytic chain scission is involved. It has already been shown in various publications that phosphites promote the degradation of PLA under acidic conditions [23,26]. Under the influence of moisture, the phosphite hydrolyzes and the resulting degradation products (including phosphorous acid) catalyze the hydrolytic ester cleavage [26].

The addition of the synergistic hydrolysis stabilizer combination, on the other hand, ensures the highest stabilizing effect. After 1000 h, the increase is only approx. 25% (12 cm^3^ 10 min^−1^ → 15 cm^3^ 10 min^−1^). A doubling of the MVR only occurs after 1750 h (21 cm^3^ 10 min^−1^). After more than 2000 h, the MVR is 30 cm^3^ 10 min^−1^ and a significant chain degradation can be observed. Compared to unstabilized PLA, degradation is delayed by approx. 1500 h. The combination consists of an aziridine-based hydrolysis inhibitor (PolyU) and calcium hydrotalcite (HTC 1) as an acid scavenger. Addition of the PolyU aziridine provides a dual effect. At first it acts as moisture scavenger by reacting with the low quantity of residual moisture present in the polymer matrix. As a result, the moisture can no longer react with the polymer chains and, therefore, these are stabilized. In addition, the aziridine also reacts with carboxylic end groups of the polymer, which prevents autocatalytic degradation. The stabilizing effect of hydrotalcite is based on an acid-regulating effect within the polymer matrix. As the rate of hydrolytic degradation depends on the pH value [42], the addition of the acid regulator reduces the rate of degradation. Furthermore, the acid-regulating effect slows down the consumption of the hydrolysis inhibitor. By combining both stabilization mechanisms in a synergistic manner, particularly extended stabilization is achieved. The specific stabilization mechanisms have already been described in more detail in a previous publication [40].

It can be concluded that only the addition of a hydrolysis stabilizer leads to an increase in thermal stability during oven aging, whereby it should be noted here that the addition of a hydrolysis stabilizer requires a significantly larger dosage. Therefore, it must be assumed that ester hydrolysis is the dominant degradation mechanism in the solid phase, even under increased thermal stress. Oxidative degradation appears to play only a subordinate role, where even the addition of antioxidants does not improve long-term thermal stability.

To ensure that hydrolytic degradation is not caused by high humidity during thermal aging, moisture content measurements of the PLA granules were carried out (Table 1). As a reference, measurements of raw PLA granules (stored in Standard climate) and PLA pellets from a 60 °C water storage were performed for comparison.

Table 1 shows the moisture content of PLA under the various aging conditions. The moisture content of PLA raw material is approx. 2600 ppm (0.26%). During oven aging at 100 °C, the moisture content is reduced to just over 100 ppm (0.01%). It can, therefore, be assumed that there is no unreasonably high moisture level during thermal aging. This low moisture content appears to be sufficient for hydrolytic chain degradation to occur at the high temperatures. In comparison, the moisture content during 60 °C water storage is approx. 11,000 ppm (1.1%).

Subsequently, the hydrolysis-stabilized compound was again aged at a temperature of 150 °C.

**Figure 9:**
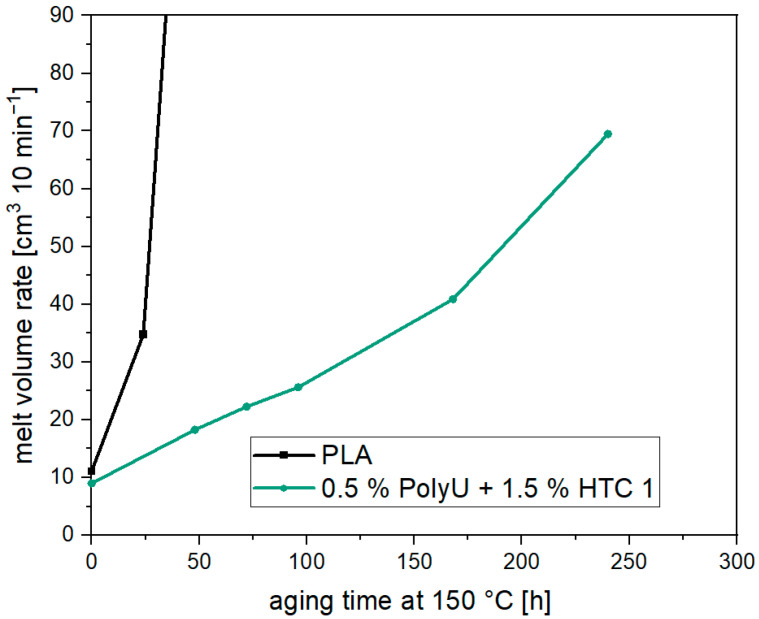
Melt volume rate over aging time at 150 °C for PLA with a hydrolysis stabilizer.

Figure 9 shows the MVR of hydrolysis-stabilized PLA at an aging temperature of 150 °C over 250 h. Compared to the unstabilized material, a significantly improved stability can be observed. Nevertheless, there is a continuous increase in the MVR even after short aging times. The MVR has already doubled after 72 h (→ 22 cm^3^ 10 min^−1^). After 240 h, the MVR is already over 70 cm^3^ 10 min^−1^. Significant chain degradation can be observed with longer aging times. The MVR of this sample also decreases later on. However, the solid phase condensation starts much later than with the unadditivated PLA. Nevertheless, it should be noted that at a temperature of 150 °C, chain degradation starts too quickly, and sufficient stability is not achieved, even with stabilizers.

## 4. Conclusions

The thermal aging behavior of PLA in the solid phase was investigated. It was shown that the degradation is not primarily caused by oxidative degradation mechanisms but is promoted to a significant extent by hydrolytic chain scission. High aging temperatures not only lead to chain degradation, but also to solid phase condensation. After a certain degree of chain degradation has occurred, the polymer chains recombine, which was demonstrated by size-exclusion-chromatography. However, this requires the formation of hydroxyl- and carboxyl-end groups that are not formed during oxidative degradation, but during hydrolytic degradation reactions, which was demonstrated via nuclear magnetic resonance spectroscopy. The hypothesis that the chain scission during aging is caused by hydrolytic degradation was confirmed by experiments with various polymer stabilizers. The addition of phenolic, phosphitic and thio-based structures, which act as stabilizers against oxidative degradation, did not lead to an increased aging stability. These structures are only effective against radical degradation mechanisms, which based on the spectroscopy tests, only play a subordinate role here. They have no effect against hydrolytic degradation, and therefore, do not increase the thermal stability during aging in the solid phase. The addition of phosphites even catalyzes chain degradation during thermal aging. This effect has already been demonstrated in the literature for hydrolytic degradation, which again confirms that this is the primary degradation mechanism here. However, the addition of a synergistic combination consisting of a hydrolysis inhibitor and an acid scavenger, which has proven to be a particularly effective stabilizer against hydrolytic degradation in previous studies, resulted in significantly improved thermal aging stability. While significant chain degradation, at an aging temperature of 100 °C, occurs after 500–600 h for unstabilized PLA, the material with the hydrolysis stabilizer remains stable for up to 2000 h. The specimens stabilized with antioxidants, on the other hand, also degrade after approx. 500–600 h. Aging at a temperature of 150 °C showed that even the addition of a hydrolysis stabilizer only had a short-term stabilizing effect and that significant degradation occurred after 250 h.

## Figures and Tables

**Table 1:** Moisture content of PLA under different conditions.

Conditions	Moisture Content/ppm
PLA batch	2601
100 °C thermal aging	110
60 °C water bath	11,080

## Data Availability

The original contributions presented in the study are included in the article, further inquiries can be directed to the corresponding author.
